# Exergy-Based Multi-Objective Optimization of an Organic Rankine Cycle with a Zeotropic Mixture

**DOI:** 10.3390/e23080954

**Published:** 2021-07-26

**Authors:** Zineb Fergani, Tatiana Morosuk, Djamel Touil

**Affiliations:** 1 Laboratory of Biomaterials and Transport Phenomena, Department of Process and Environmental Engineering, University of Medea, Medea 26000, Algeria; ferganizineb@gmail.com; 2 Institute for Energy Engineering, Technische Universität Berlin, 10587 Berlin, Germany; 3 Department of Process Engineering, Faculty of Technology, University of Blida, Blida 090000, Algeria; touillepa@gmail.com

**Keywords:** organic Rankine cycle, zeotropic mixture, exergy-based analysis, multi-objective optimization

## Abstract

In this paper, the performance of an organic Rankine cycle with a zeotropic mixture as a working fluid was evaluated using exergy-based methods: exergy, exergoeconomic, and exergoenvironmental analyses. The effect of system operation parameters and mixtures on the organic Rankine cycle’s performance was evaluated as well. The considered performances were the following: exergy efficiency, specific cost, and specific environmental effect of the net power generation. A multi-objective optimization approach was applied for parametric optimization. The approach was based on the particle swarm algorithm to find a set of Pareto optimal solutions. One final optimal solution was selected using a decision-making method. The optimization results indicated that the zeotropic mixture of cyclohexane/toluene had a higher thermodynamic and economic performance, while the benzene/toluene zeotropic mixture had the highest environmental performance. Finally, a comparative analysis of zeotropic mixtures and pure fluids was conducted. The organic Rankine cycle with the mixtures as working fluids showed significant improvement in energetic, economic, and environmental performances.

## 1. Introduction

The organic Rankine cycle (ORC) has a large potential for electricity generation from heat sources with relatively low temperatures such as geothermal, solar, biomass, and waste industrial heat. Different aspects of ORCs have been studied intensively. In ORC, the selection of a working fluid is an essential factor that affects the cycle’s performances [1] including the economic and environmental aspects.

**Figure 1:**
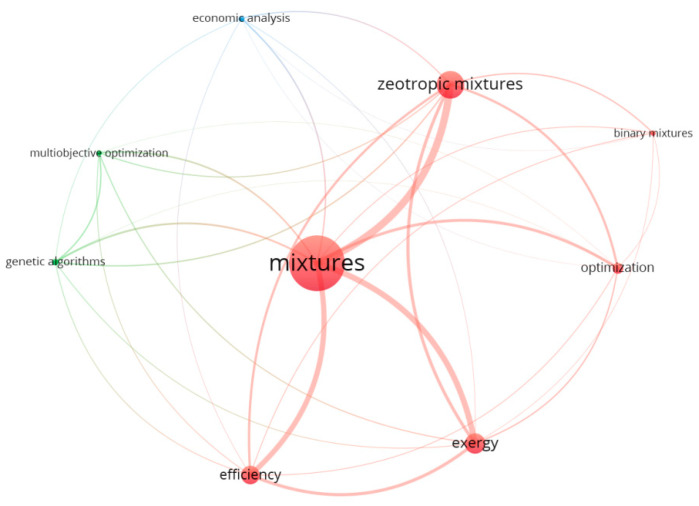
Co-occurrence and links among the keywords (by VOSviewer).

For the bibliometric analysis of the state-of-the-art developments in the field of multi-objective optimization applied for ORC, the Scopus database (April 2021) was used with the following algorithm. The initial keyword “ORC” was used with the following equivalents: “Organic Rankine cycle” = “Organic Rankine cycle (ORC)” = “Organic Rankine cycles” = “ORCs”. The only publications were considered if they met the following criteria: (a) in English; (b) in an international journal, and (c) in the proceedings of an international conference. As a result, 3058 publications were selected. Through the application of filter “optimization”, the number of publications was reduced to 2228. To describe state-of-the-art developments in the field of authors’ research, a second step of filters was applied. Finally, 456 papers were selected, with at least one of the following keywords: “working fluids”, “economic analysis”, “genetic algorithm”, “binary mixture”, and “multiobjective optimization”. To identify the links among the keywords, the software VOSviewer [2] was employed. Figure 1 shows the co-occurrence and links among the keywords. The evaluation of the obtained results demonstrates that within “multiobjective optimization”, only thermodynamic and economic variables were considered. None of the evaluated papers addressed the evaluation of ORC using thermodynamic, economic, and environmental aspects simultaneously (particularly, based on the concept of exergy) as well as included in the optimization. The genetic algorithm approach was applied in a larger number of papers than “multiobjective optimization”.

The detailed literature review of the most representative papers is as follows. Note that in the below mentioned studies, the research results for the ORC with one-component working fluids are reported (not included in Figure 1). For example, the thermodynamic analysis and optimization of ORC performance with one-component working fluids are discussed in [3,4,5,6,7]. Several studies have evaluated the ORC using different performance criterions, such as energetic, economic, and environmental, using exergy tools [8,9]. Exergy can be combined with economic analysis and an environmental assessment; these combinations are called exergoeconomic and exergoenvironmental analysis, or exergy-based methods. In [4], a parametric optimization of an ORC using R123, R245fa, and isobutane as working fluids has been performed from the perspectives of thermodynamic and economic. The exergetic performance of an ORC with high critical temperature working fluids using genetic algorithm optimization was investigated in [10]. Thermodynamics and exergoeconomics performances of ORC with several one-component working fluids were investigated and compared with those of the Kalina cycle and trilateral power cycle. The obtained results reveal that the ORC system is the most recommended for generating power among the two cycles studied from the perspective of economics [11]. In [12], multi-objective optimization of an ORC with cyclohexane, benzene, and toluene as the working fluids using the exergy, exergoeconomic, and exergoenvironmental approaches has been reported.

Within Figure 1, the following papers were included. The mismatch of the isothermal phase change line for evaporators and condensers and the heat source and sink lines led to large irreversibility in two main heat exchangers [13]. Similar to refrigeration applications, different mixtures were discussed for use as the working fluids for ORC. Zeotropic mixtures have the temperature glide in the two-phase zone; therefore, they can be selected in order to bring the temperature profiles closer in the heat exchangers [14]. The performance of the ORC using different zeotropic mixtures on the basis of thermodynamics and thermoeconomics is discussed in [1]. The results reveal that the ORC using the mixture, generally, demonstrates a low economic performance. The thermodynamic and thermoeconomic comparison analysis of an ORC system with one-component working fluids and mixtures are reported in [13]. The considered one-component working fluids are high and low critical temperatures. The obtained results demonstrate that the thermoeconomic performance of working fluids with high critical temperatures is better than those with low critical temperatures. A comparative study of one-component working fluids and mixtures for ORC, from the energy and exergy viewpoints, was reported in [15]. They reported that evaluated mixtures have lower efficiency than one-component working fluids. In [16], performance analysis and parametric optimization of several zeotropic mixtures for an ORC using an exergy approach were performed; the mixture R245fa/R600a (0.9/0.1) was reported as most advantageous. Thermodynamic analysis and multi-objective optimization for various configurations of ORC using zeotropic mixtures were performed in [17]. The results indicated that zeotropic mixtures showed a higher performance than one-component working fluids. A comparison of thermodynamic and exergoeconomic performances for supercritical CO_2_ recompression cycle combined with regenerative organic Rankine cycle using the zeotropic mixture as working fluid was reported in [18]. In [19], a complex thermo-economic–environmental optimization and advanced exergy analysis were applied for a dual-loop organic Rankine cycle (DORC) using zeotropic mixtures. The payback period was selected as an economic evaluation criteria and annual CO_2_ emission reduction as an environmental evaluation criterion. Higher performance was observed for the mixtures as working fluid of ORC. Both criteria, payback period and annual CO_2_ emission reduction, could not be linked to the exergy variables (therefore, [19] was not included in Figure 1).

As it can be seen from the literature review, there are valuable research works that address the use of mixtures as working fluids for ORC. However, to the best of the authors’ knowledge, there are no research results regarding the exergoeconomic and exergoenvironmental evaluation of ORC with mixtures as the working fluids. The main purpose of this study was to evaluate an ORC system with a zeotropic mixture as the working fluid for power generation using waste heat from a cement plant. The zeotropic mixtures under this study were toluene/cyclohexane and toluene/benzene.

For the evaluation, exergy-based methods were applied, and for the optimization, a multi-objective optimization approach was used.

## 2. System Description

**Figure 2:**
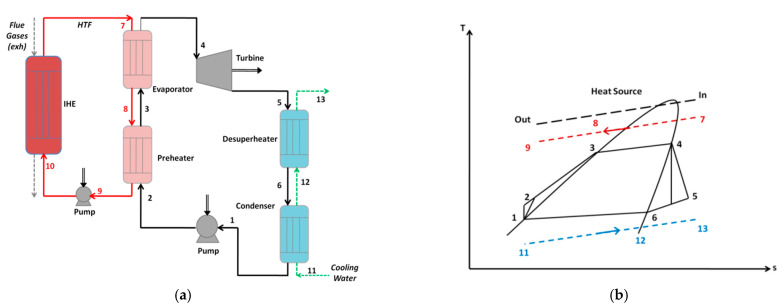
Flow diagram (**a**) and temperature–entropy diagram (**b**) for the ORC process.

A flow diagram of the proposed ORC system is given in Figure 2a. The system consists of four components: a generator as a combination of a preheater and an evaporator, a turbine, a condenser, and a pump. The processes within the ORC are illustrated in the temperature–entropy (*T–s*) diagram in Figure 2b. The pump pressurizes the working fluid (state 2) to the evaporator pressure. The working fluid is heated and evaporated by absorption of the heat from a heat source. The working fluid vapor (state 4) flow enters the turbine and generates the shaft work. The low pressure vapor (state 5) leaves the turbine to the condenser.

In the evaluated ORC system, the heat source is the exhaust gas with a temperature of 350 °C from a technological process [20]. The utilization of waste technological heat requires the use of intermediate working fluid—thermal oil.

The considered working fluids, which are zeotropic mixtures that have high critical and boiling temperatures, are also characterized by “dry” properties and high thermodynamic performance for ORC application [21]. This choice was based on the slope of the saturated vapor line for the working fluid on a *T–s* diagram and the temperature level of the heat source [6,22]. The mixtures of toluene with cyclohexane and toluene with benzene at different concentrations are discussed in the present study as well.

## 3. System Modeling and Analysis

The ORC system model was developed using MATLAB software. Refprop software and equations [23] were used for calculating the properties of the working fluids. Dowtherm $\dot{Q}$ was chosen as an intermediate heat transfer fluid. All properties were calculated using the equations in [24]. The simulation model was developed with the following assumptions: (a) steady-state operation conditions, (b) pressure drop and exergy losses within heat exchangers are neglected, and (c) the mass fraction shift of a zeotropic mixture is neglected in the case of each composition.

### 3.1. Thermodynamic Modeling

According to Figure 2, the thermodynamic model of the ORC is described below. All components were simulated under the assumption of adiabatic operation conditions.

- Pumps
  (1) $${\dot{W}}_{p,ORC}={\dot{m}}_{wf}\left(h_{2}-h_{1}\right)$$
  (2) $${\dot{W}}_{p,HTF}={\dot{m}}_{HTF}\left(h_{10}-h_{9}\right)$$
- Turbine
  (3) $${\dot{W}}_{t}={\dot{m}}_{wf}\left(h_{4}-h_{5}\right)$$
- Heat exchangers

The heat balance equations in the intermediate heat exchanger (IHE), evaporator (evp), preheater (pre), desuperheater (desp), and condenser (con) can be, respectively, expressed as:

(4) $${\dot{Q}}_{IHE}={\dot{m}}_{HTF}c_{p,HTF}\left(T_{7}-T_{10}\right)$$

(5) $${\dot{Q}}_{evp}={\dot{m}}_{wf}\left(h_{4}-h_{3}\right)={\dot{m}}_{HTF}c_{p.HTF}\left(T_{7}-T_{8}\right)$$

(6) $${\dot{Q}}_{pre}={\dot{m}}_{wf}\left(h_{3}-h_{2}\right)={\dot{m}}_{HTF}c_{p,HTF}\left(T_{8}-T_{9}\right)$$

(7) $${\dot{Q}}_{dusp}={\dot{m}}_{wf}\left(h_{5}-h_{6}\right)={\dot{m}}_{w}c_{p,w}\left(T_{13}-T_{12}\right)$$

(8) $${\dot{Q}}_{con}={\dot{m}}_{wf}\left(h_{6}-h_{1}\right)={\dot{m}}_{w}c_{p,w}\left(T_{12}-T_{11}\right)$$

All heat exchanges are a shell-and-tube type.

(9) $${\dot{Q}}_{k}=U_{k}A_{k}LMDT_{k}$$

where the size of these components (i.e., heat transfer surface (*A*)) are calculated with the help of the heat transfer coefficient correlations *U_k_* [25,26] and the logarithmic mean temperature difference method *LMTD_k_*.

### 3.2. Exergy Analysis

The exergy analysis was performed using the approach of “exergy of fuel, ${\dot{E}}_{F,k}$” and “exergy of product, ${\dot{E}}_{P,k}$”. The value of irreversibilities was expressed through exergy destruction, ${\dot{E}}_{D,k}$. The exergy balance for each system component is written as [27]:

(10) $${\dot{E}}_{F,k}={\dot{E}}_{P,k}+{\dot{E}}_{D,k}$$

### 3.3. Exergoeconomic Analysis

In order to proceed with an exergoeconomic analysis, a cost balance is supposed to be written for the *k*th component of ORC. Where necessary, the auxiliary equations should be added to the corresponding cost balance [27] using the P-rule and/or F-rule:

(11) $$\sum \;{\dot{C}}_{out}=\sum \;{\dot{C}}_{in}+{\dot{Z}}_{k}$$

With the exergy costing principle $\dot{C}=c\;\dot{E}$. The term ${\dot{Z}}_{k}$ represents the total capital investment cost rate; it was determined according to cost equations reported in [12].

Cost balances for each component must be resolved simultaneously. A linear equations system was developed by combining Equation (11) with the auxiliary equations:

(12) $$\left[{\dot{E}}_{k}\right]\times \left[c_{k}\right]=\left[{\dot{Z}}_{k}\right]$$

**Figure 3:**
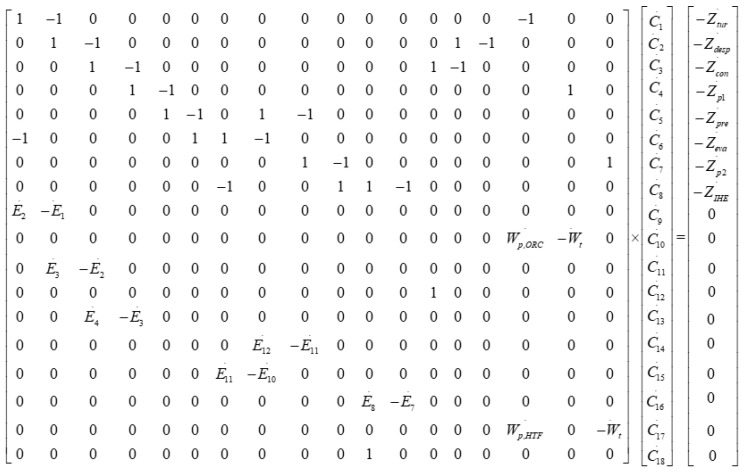
Matrix of the cost equations.

The matrix form of the cost equations is given in Figure 3.

### 3.4. Exergoenvironmental Analysis

The methodology of exergoenvironmental is similar to the exergoeconomic analysis [27]. Exergoenvironmental analysis combines exergy analysis and LCA. Environmental balances can be written as follows:

(13) $$\sum \;{\dot{B}}_{out}=\sum \;{\dot{B}}_{in}+{\dot{Y}}_{k}$$

Correlations were developed for calculating the environmental impact of the components $\left({\dot{Y}}_{k}\right)$ in the construction period. The LCA was conducted according to Eco-indicator 99 [28].

A linear equations system was developed by combining Equation (13) with the auxiliary equations:

(14) $$\left[{\dot{E}}_{k}\right]\times \left[b_{k}\right]=\left[{\dot{Y}}_{k}\right]$$

**Figure 4:**
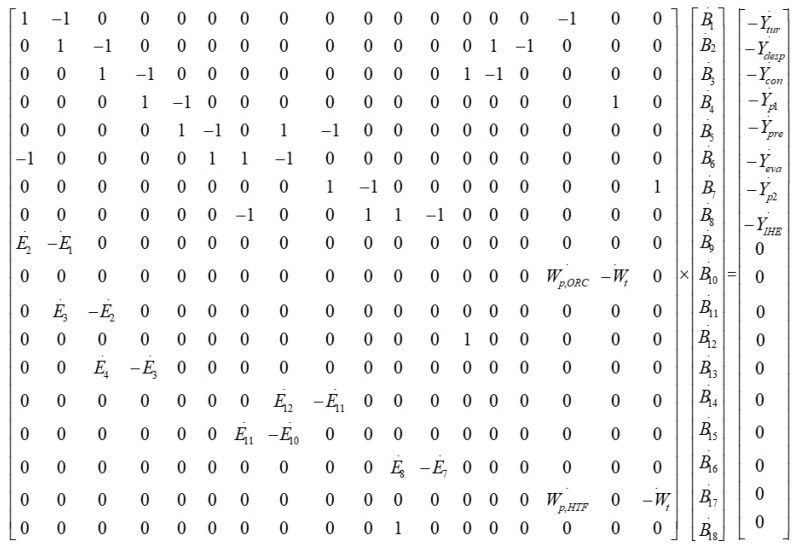
Matrix of the environmental impact equations.

Figure 4 shows the matrix formulation of the environmental impact equations. Table 1 represents all exergy, cost, and environmental balance equations for different components of the evaluated ORC system.

## 4. System Optimization

The ORC system was optimized using a multi-objective approach based on the particle swarm algorithm [29]. Pareto frontier was supposed to be obtained for the total system. The following three objective functions were considered in this study:

- Exergy efficiency
  (15) $$E_{sys}=\frac{{\dot{W}}_{net}}{{\dot{E}}_{exh,\;in}}=\frac{{\dot{W}}_{t}-\left({\dot{W}}_{P,ORC}+{\dot{W}}_{P,HTF}\right)}{{\dot{E}}_{exh,in}}$$
- Cost per exergy unit of the power generated
  (16) $$c_{p,sys}=\frac{{\dot{C}}_{net}}{{\dot{W}}_{net}}=\frac{c_{P,t}{\dot{W}}_{net}}{{\dot{W}}_{net}}$$
- Environmental impact of the power generated
  (17) $$b_{p,sys}=\frac{{\dot{B}}_{net}}{{\dot{W}}_{net}}=\frac{b_{P,t}{\dot{W}}_{net}}{{\dot{W}}_{net}}$$

## 5. Results and Discussion

The used ORC model was validated using the reported data [30] for the basic ORC system with the R245fa/R600azeotropic mixture as the working fluid. The temperature and the mass flow rate of the heat source were set as 120 °C and 1 kg/s. The pinch temperature difference in the evaporator and the condenser were considered to be 10 °C and 5 °C, respectively. The turbine efficiency and pump efficiency were assumed to be 85% and 65%, respectively. As shown in Table 2, the present results and data from [30] are in good agreement.

### 5.1. Parametric Study

In order to investigate the effect of certain parameters on the ORC cycles performances, a parametric study was carried out. The key input parameters and the underlying assumptions to simulate the ORC are provided in Table 3.

**Figure 5:**
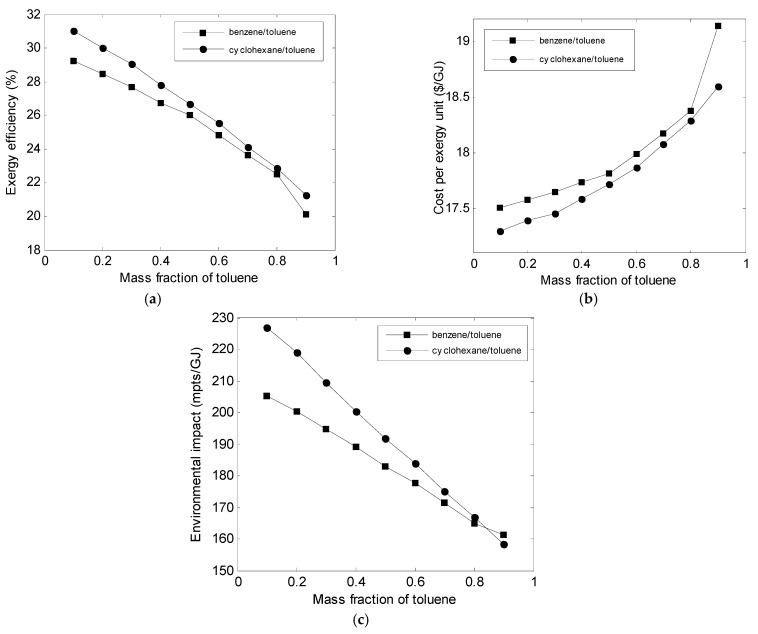
Variations of (**a**) exergy efficiency, (**b**) cost, and (**c**) environmental impact with toluene mass fraction.

The effect of the mass fraction of working fluid on the ORC performances is shown in Figure 5. For the mixtures of cyclohexane/toluene and benzene/toluene, the exergy efficiency decreased with the increasing mass fraction of toluene. The cost per unit of exergy for both mixtures increased with the increase in the mass fraction of toluene.

According to the results of the parametric study reported in [12], ORC using cyclohexane and benzene as pure fluids was more effective compare to toluene in terms of thermodynamic and economics. Increasing the mass fraction of toluene will degrade the exergetic and exergoeconomic performances of the ORC system. In addition, the environmental impact decreased as the mass fraction of toluene increased for both mixtures (Figure 5). This is because the exergoenvironmental performance of ORC with toluene as working fluid was better than that of ORC with cyclohexane and benzene as pure fluids [12].

**Figure 6:**
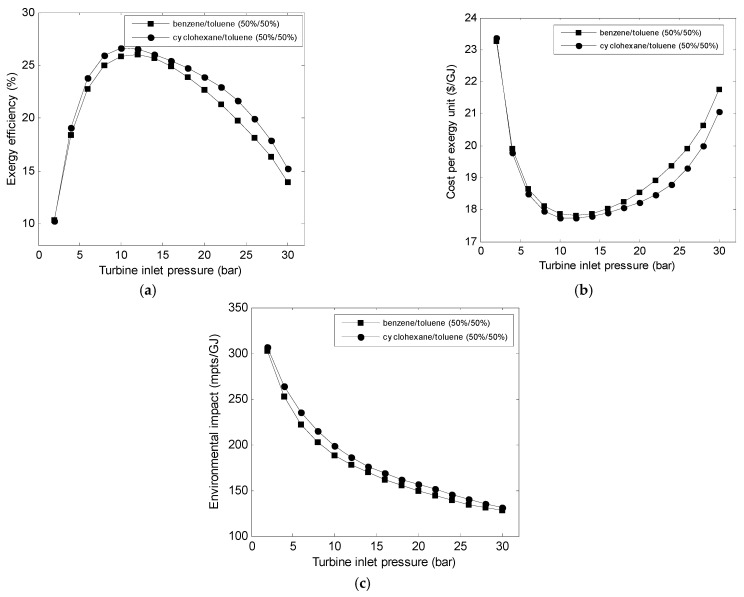
Variations in the (**a**) exergy efficiency, (**b**) cost, and (**c**) environmental impact with turbine inlet pressure.

Figure 6 shows the variation in the objective functions with turbine inlet pressure for working fluids. The exergy efficiency (Figure 6a) was maximized and cost per exergy unit (Figure 6b) minimized at a special value of turbine inlet pressure, while the environmental impact decreased with the increase in the turbine inlet pressure. These results exhibit the same characteristics as those shown in the previous work [12]. Figure 6 shows that the best exergetic and exergoeconomic performances were observed for the cyclohexane /toluene mixture, while the best exergoenvironmental performance was for the benzene/toluene mixture.

**Figure 7:**
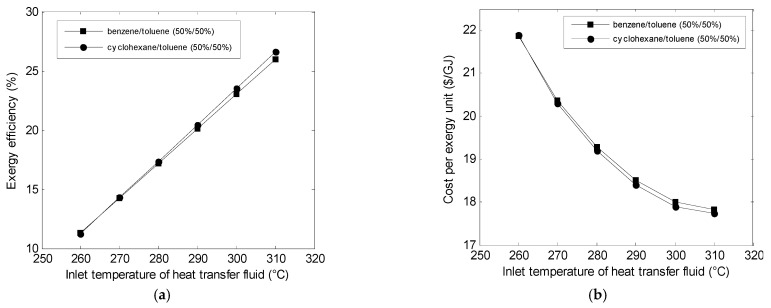

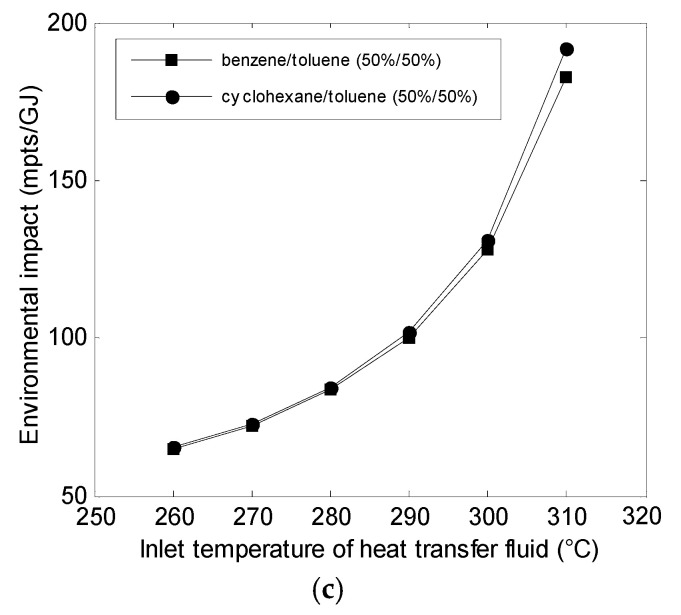
Variations in the (**a**) exergy efficiency, (**b**) cost, and (**c**) environmental impact with heat transfer fluid temperature.

Variations in the performances of the ORC cycle with the heat transfer fluid temperature were given in Figure 7 for both mixtures. It can be seen that as the heat transfer fluid temperature increased, the exergy efficiency and the environmental impact increased. On the other hand, the increase in the heat transfer fluid temperature caused a decrease in the cost. Figure 7 also indicates that when the temperature was below 270 °C, both mixtures offerred the same performance.

### 5.2. Optimization Results

A parametric optimization was conducted using the MOPSO (multi-objective particle swarm optimizer) algorithm. Particle swarm optimization is one of the most efficient evolutionary optimization algorithms widely used to resolve multi-objective optimization problems. This technique is based on the evolution of a population of solutions called particles that move within the search space. The basic parameters of the algorithm are specified according to the values presented in [12].

**Figure 8:**
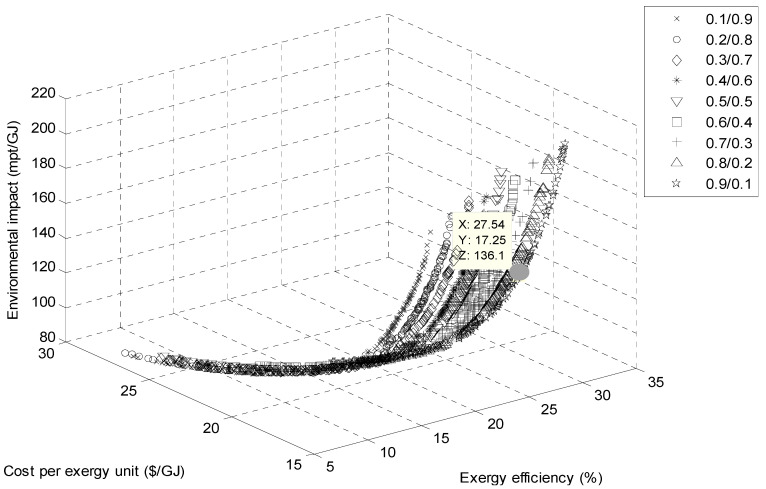
Pareto front for ORC with the cyclohexane/toluene mixtures.

**Figure 9:**
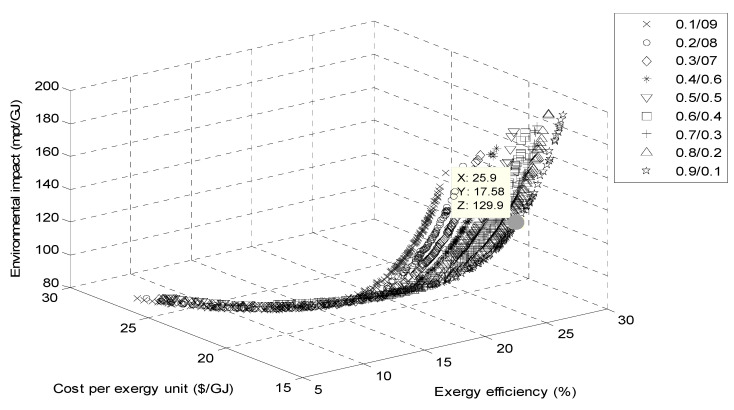
Pareto front for ORC with the benzene/toluene mixtures.

Figure 8 and Figure 9 show the Pareto frontier of the multi-objective optimization using cyclohexane/toluene and benzene/ toluene at different mass fractions. All Pareto frontier points are potentially an optimum solution. Therefore, one optimal solution must be selected.

In the present study, the final optimum design point and the optimal zeotropic mixture were selected through a fuzzy-based mechanism [31]. Thermodynamic properties and optimization results for the zeotropic mixture are indicated in Table 4 and Table 5. It should be noted that the best results were found for both mixtures with a concentration of 0.9/0.1. Referring to Table 5, the exergy efficiency of the ORC using cyclohexane/toluene was higher than that of using benzene/toluene. This is because the cyclohexane/toluene mixture exhibited the highest turbine inlet pressure. As mentioned in a previous work, a higher turbine inlet pressure working fluid provided the highest values of power and exergy efficiency [12]. On the other hand, a cyclohexane/toluene mixture provides the best result from the viewpoint of exergoeconomics, while the best exergoenvironmental performance was obtained for the benzene/toluene mixture.

When comparing performances of pure and mixture fluids, it can be found that the zeotropic mixtures exhibited low turbine inlet pressure, which may be desirable because high pressures lead to mechanical constraints and, therefore, expensive equipment may be needed [32]. It can also be seen that the exergetic performances of zeotropic mixtures were slightly higher than pure cyclohexane and pure benzene.

Compared to pure toluene, a significant increase in the exergy efficiency was observed; the exergetic performance improved 53.0% and 43.5% when the toluene was mixed with cyclohexane and benzene, respectively.

From Table 5, we also can see that the zeotropic mixtures of cyclohexane/toluene and benzene/toluene had the best exergoeconomic performances in comparison with pure fluids. The improvement in exergoeconomic performance for cyclohexane and benzene when they were mixed with toluene was 4.9%, while the improvement was 14.6% and 13.0% if toluene was mixed with cyclohexane and benzene. On the other hand, the zeotropic mixtures also showed a significant improvement in exergoenvironmental performance. The improvement was 8.2% and 10.2% for cyclohexane and benzene, respectively, while the improvement was 14.8% and 18.8% for toluene if it was mixed with cyclohexane and benzene, respectively.

In Table 6, the results obtained from the exergy, exergoeconomic and exergoenvironmental analyses are reported. The results of exergy analysis indicate that the highest exergy destruction for both working fluids occurred in the heat exchangers.

Based on the exergoeconomic analysis, the heat exchangers had the highest cost rate (${\dot{C}}_{D}$) as a result of high exergy destruction, while the larger investment cost ($\dot{Z}$) corresponded to the condenser–desuperheater assembly and the turbine. These two components are the most important from an exergoeconomic viewpoint, as they have a higher ($\dot{Z}$ + ${\dot{C}}_{D}$) value.

In terms of exergoenvironmental analysis, the heat exchangers had a larger environmental impact (${\dot{B}}_{D}$) among the ORC components. The results also indicate that much of the component-related environmental impact ($\dot{Y}$) came from these components. According to the value of ($\dot{Y}$ + ${\dot{B}}_{D}$), more attention is needed on these components.

## 6. Conclusions

In this research paper, exergy, exergoeconomic, and exergoenvironmental analyses were applied in order to evaluate the performance of the ORC system using zeotropic mixtures as working fluids. Parametric studies were carried out to evaluate the influence of operational parameters on the exergetic, economic, and environmental performances of the evaluated system. Multi-objective optimization was applied to ensure the optimum performances of the ORC system with two zeotropic mixtures (cyclohexane/toluene and benzene/toluene). A comparison between performances of pure and mixture working fluids was discussed and the following conclusions were obtained:

- The application of zeotropic mixtures as a working fluid for ORC led to an increase in exergetic, exergoeconomic, and exergoenvironmental performances compared to using their pure constituents;
- The heat exchangers were the most important ORC system components based on the exergy, exergoeconomic, and exergoenvironmental points;
- The mass fraction of working fluids within a zeotropic mixture, turbine inlet pressure, and heat transfer fluid temperature had a significant effect on the exergetic, exergoeconomic, and exergoenvironmental performance of the ORC system;
- Cyclohexane/toluene (mass fraction 90/10) and benzene/toluene (mass fraction 90/10) are recommended as the optimal mixtures for the selected operating conditions;
- The mixture of cyclohexane and toluene will be a better choice only if energetic and economic criterions are considered. However, the mixture benzene/toluene is a beneficial choice to fulfill the environmental criteria.

## Figures and Tables

**Table 1:** Exergy, cost, and environmental balances within ORC system components.

Component	Fuel	Product	Cost Balances and Auxiliary Equations	Environmental Balances and Auxiliary Equations
Turbine	${\dot{E}}_{4}-{\dot{E}}_{5}$	${\dot{W}}_{t}$	${\dot{C}}_{4}+{\dot{Z}}_{t}={\dot{C}}_{5}+{\dot{C}}_{Wt}$ $c_{{\dot{W}}_{p}}=c_{{\dot{W}}_{tur}}$ $Z=6000\;{\dot{W}}_{tur}^{0.7}$	${\dot{B}}_{4}+{\dot{Y}}_{t}={\dot{B}}_{5}+{\dot{B}}_{Wt}$ $b_{{\dot{W}}_{p}}=b_{{\dot{W}}_{tur}}$ $\dot{Y}=\left(M\times \omega_{steel}\right)/\left(\tau \times n\right)$
Preheater	${\dot{E}}_{8}-{\dot{E}}_{9}$	${\dot{E}}_{3}-{\dot{E}}_{2}$	${\dot{C}}_{2}+{\dot{C}}_{8}+{\dot{Z}}_{pre}={\dot{C}}_{3}+{\dot{C}}_{9}$ $c_{8}=c_{9}$ $Z=10,000+324\;A^{0.91}$	${\dot{B}}_{2}+{\dot{B}}_{8}+{\dot{Y}}_{pre}={\dot{B}}_{3}+{\dot{B}}_{9}$ $b_{8}=b_{9}$ $\dot{Y}=(\rho_{steel}\times \delta \times \omega_{steel}\times A)/\left(\tau \times n\right)$
Evaporator	${\dot{E}}_{7}-{\dot{E}}_{8}$	${\dot{E}}_{4}-{\dot{E}}_{3}$	${\dot{C}}_{3}+{\dot{C}}_{7}+{\dot{Z}}_{eva}={\dot{C}}_{4}+{\dot{C}}_{8}$ $c_{7}=c_{8}$ $Z=10,000+324\;A^{0.91}$	${\dot{B}}_{3}+{\dot{B}}_{7}+{\dot{Y}}_{eva}={\dot{B}}_{4}+{\dot{B}}_{8}$ $b_{7}=b_{8}$ $\dot{Y}=(\rho_{steel}\times \delta \times \omega_{steel}\times A)/\left(\tau \times n\right)$
Desuperheater	${\dot{E}}_{5}-{\dot{E}}_{6}$	${\dot{E}}_{13}-{\dot{E}}_{12}$	${\dot{C}}_{5}+{\dot{C}}_{12}+{\dot{Z}}_{desup}={\dot{C}}_{6}+{\dot{C}}_{13}$ $c_{5}=c_{6}$ $Z=10,000+324\;A^{0.91}$	${\dot{B}}_{5}+{\dot{B}}_{12}+{\dot{Y}}_{desup}={\dot{B}}_{6}+{\dot{B}}_{13}$ $b_{5}=b_{6}$ $\dot{Y}=(\rho_{steel}\times \delta \times \omega_{steel}\times A)/\left(\tau \times n\right)$
Condenser	${\dot{E}}_{6}-{\dot{E}}_{1}$	${\dot{E}}_{12}-{\dot{E}}_{11}$	${\dot{C}}_{6}+{\dot{C}}_{11}+{\dot{Z}}_{con}={\dot{C}}_{1}+{\dot{C}}_{12}$ $c_{6}=c_{1}c_{11}=0\;\;$ $Z=10,000+324\;A^{0.91}$	${\dot{B}}_{6}+{\dot{B}}_{11}+{\dot{Y}}_{con}={\dot{B}}_{1}+{\dot{B}}_{12}$ $b_{6}=c_{1}b_{11}=0$ $\dot{Y}=(\rho_{steel}\times \delta \times \omega_{steel}\times A)/\left(\tau \times n\right)$
IHE	${\dot{E}}_{exh,in}-{\dot{E}}_{exh,out}$	${\dot{E}}_{7}-{\dot{E}}_{10}$	${\dot{C}}_{exh,in}+{\dot{C}}_{10}+{\dot{Z}}_{IHE}={\dot{C}}_{exh,out}+{\dot{C}}_{7}$ $c_{exh,in}=c_{exh,out}$ $Z=10,000+324\;A^{0.91}$	${\dot{B}}_{exh,in}+{\dot{B}}_{10}+{\dot{Y}}_{IHE}={\dot{B}}_{exh,out}+{\dot{B}}_{7}$ $b_{exh,in}=b_{exh,out}$ $\dot{Y}=(\rho_{steel}\times \delta \times \omega_{steel}\times A)/\left(\tau \times n\right)$
Pump_ORC_	${\dot{W}}_{P,ORC}$	${\dot{E}}_{2}-{\dot{E}}_{1}$	${\dot{C}}_{1}+{\dot{C}}_{{\dot{W}}_{p}}+{\dot{Z}}_{p}={\dot{C}}_{2}$ $c_{{\dot{W}}_{p}}=c_{{\dot{W}}_{t}}$ $Z=422{\dot{W}}_{p}^{0.71}\left[1.41+1.41\left(\frac{1-0.8}{1-{ƞ}_{p}}\right)\right]$	${\dot{B}}_{1}+{\dot{B}}_{{\dot{W}}_{p}}+{\dot{Y}}_{p}={\dot{B}}_{2}$ $b_{{\dot{W}}_{p}}=b_{{\dot{W}}_{t}}$ $\dot{Y}=\left(M\times \omega_{steel}\;\right)/\left(\tau \times n\right)$
Pump_HTF_	${\dot{W}}_{P,HTF}$	${\dot{E}}_{10}-{\dot{E}}_{9}$	${\dot{C}}_{9}+{\dot{C}}_{{\dot{W}}_{p,HTF}}+{\dot{Z}}_{p,HTF}={\dot{C}}_{10}$ $c_{{\dot{W}}_{p,HTF}}=c_{{\dot{W}}_{t}}$ $Z=422{\dot{W}}_{p}^{0.71}\left[1.41+1.41\left(\frac{1-0.8}{1-{ƞ}_{p}}\right)\right]$	${\dot{B}}_{9}+{\dot{B}}_{{\dot{W}}_{p,HTF}}+{\dot{Y}}_{p,HTF}={\dot{B}}_{10}$ $b_{{\dot{W}}_{p,HTF}}=b_{{\dot{W}}_{t}}$ $\dot{Y}=\left(M\times \omega_{steel}\right)/\left(\tau \times n\right)$

**Table 2:** Model verification results.

	Performances	Results of This Study	Results from [30]
$T_{con}=25$ °C *R245fa/R600a* (0.413/0.569)	${\dot{W}}_{net}$ (kW) ${ƞ}_{cycle}$ (%)	36.10 11.11	36.73 11.12
$T_{con}=30$ °C *R245fa/R600a* (0.437/0.563)	${\dot{W}}_{net}$ (kW) ${ƞ}_{cycle}$ (%)	32.21 10.50	32.52 10.51
$T_{con}=35$ °C *R245fa/R600a* (0.443/0.557)	${\dot{W}}_{net}$ (kW) ${ƞ}_{cycle}$ (%)	28.62 9.90	28.92 9.91

**Table 3:** A summary of the major parameters for the simulation of ORC [12].

Parameter	Value
$T_{0}$ (°C)	25
$p_{0}$ (bar)	1.01
${\dot{m}}_{exh}$ (kg/s)	48.34
${\dot{m}}_{HTF}$ (kg/s)	25.00
$T_{exh,in}$ (°C)	350
$T_{7}$ (°C)	310
$\Delta T_{eva}$ (°C)	20
$p_{4}$ (bar)	12.00
$p_{1}$ (bar)	1.02
${ƞ}_{t}$	0.85
${ƞ}_{p}$	0.70

**Table 4:** Thermodynamic proprieties of mixture fluids under optimum conditions.

	Cyclohexane/Toluene (90%/10%)				Benzene/Toluene (90%/10%)			
	*T* (°C)	*p* (bar)	$\dot{m}$ (kg/s)	*h* (kJ/kg)	*T* (°C)	*p* (bar)	$\dot{m}$ (kg/s)	*h* (kJ/kg)
1	81.7	1.02	19.70	−0.936	81.2	1.02	18.39	−0.814
2	82.3	13.9	19.70	1.2849	81.7	12.39	18.39	0.9407
3	204.2	13.91	19.70	299.43	193.1	12.39	18.39	235.93
4	204.9	13.91	19.70	541.09	194.2	12.39	18.39	529.47
5	140.1	1.02	19.70	454.56	119.4	1.02	18.39	442.89
6	82.8	1.02	19.70	356.65	82.7	1.01	18.39	392.43
7	299.5	-	25	757.74	298.4	-	25	754.95
8	224.2	-	25	567.28	213.1	-	25	539.04
9	131.3	-	25	332.29	144.7	-	25	366.19
10	132.3	-	25	334.82	145.7	-	25	368.72
11	25.0	1.02	50.33	104.92	25.0	1.02	52.17	104.92
12	58.5	1.02	50.33	244.89	58.15	1.02	52.17	243.51
13	67.6	1.02	50.33	283.24	62.4	1.02	52.17	261.29

**Table 5:** Thermodynamic proprieties of mixture fluids under optimum conditions.

Parameters	Cyclohexane/ Toluene (90/10)	Benzene/ Toluene (90/10)	Cyclohexane [12]	Benzene [12]	Toluene [12]
*p_4_* (bar)	13.91	12.39	15.12	14.43	12.96
*T_7_* (°C)	299.5	298.4	296.8	297.1	310.0
*ΔT_eva_* (°C)	20.0	20.0	20.0	20.0	21.1
*ΔT_con_* (°C)	24.3	24.6	37.0	34.6	26.1
${\dot{E}}_{exh,in}$ (MW)	5.799	5.799	5.799	5.799	5.799
${\dot{E}}_{exh,out}$ (MW)	1.272	1.567	1.383	1.736	2.431
$\varepsilon_{sys}$ (%)	27.5	25.9	27.1	25.6	18.0
*c_p,sys_* ($/GJ)	17.25	17.58	18.14	18.49	20.21
*b_p,sys_* (mpts/GJ)	136	130	148	144	160

**Table 6:** Exergetic, exergoeconomic, and exergoenvironmental analysis results.

	Turbine	Preheater– Evaporator Assembly	Desuperheater–Condenser Assembly	Pump_ORC_	Pump_HTO_	IHE
**Cyclohexane/Toluene**						
${\dot{E}}_{D}$ (kW)	219.4	623.5	982.2	7.3	46.6	463.2
${\dot{C}}_{D}$ ($/h)	2.37	4.45	10.62	0.46	2.89	2.39
$\dot{Z}$ ($/h)	85.03	4.49	354.70	1.41	1.84	23.36
$\dot{Z}+{\dot{C}}_{D}$ ($/h)	87.40	8.94	365.32	1.87	4.73	25.75
${\dot{B}}_{D}$ (mPts/h)	93	213	418	4	23	139
$\dot{Y}$ (mPts/h)	17	67	13,643	4	6	1358
$\dot{Y}+{\dot{B}}_{D}$ (mPts/h)	110	280	14,061	8	28	1497
**Benzene/Toluene**						
${\dot{E}}_{D}$ (kW)	216.4	684.6	876.0	5.4	45.1	435.6
${\dot{C}}_{D}$ ($/h)	2.36	4.89	9.53	0.34	2.85	2.20
$\dot{Z}$ ($/h)	81.09	3.95	224.41	1.14	1.84	21.40
$\dot{Z}+{\dot{C}}_{D}$ ($/h)	83.45	8.84	233.94	1.48	4.69	23.60
${\dot{B}}_{D}$ (mPts/h)	87	215	352	2	21	120
$\dot{Y}$ (mPts/h)	15	53	8214	3	6	1164
$\dot{Y}+{\dot{B}}_{D}$ (mPts/h)	102	268	8566	5	27	1284

## Data Availability

Not Applicable.

## Nomenclature

*A*	area (m^2^)
$\dot{B}$	environmental impact rate (Pts/h)
*b*	environmental impact per unit of exergy (Pts/GJ)
$\dot{C}$	cost rate ($/h)
*c*	cost per exergy unit ($/GJ)
*c_p_*	heat capacity (kJ/kg K)
$\dot{E}$	exergy rate (kW)
*h*	specific enthalpy (kJ/kg)
*M*	weight of equipment (kg)
$\dot{m}$	mass flow rate (kg/s)
*p*	pressure (bar)
*Q*	heat flow rate (kW)
*T*	temperature (°C, K)
*U*	overall heat transfer coefficient (W/m^2^ K)
$\dot{W}$	power (kW)
$\dot{Y}$	component-related environmental impact (Pts/h)
$\dot{Z}$	capital investment cost rate ($/h)
**Abbreviations**	
HTF	heat transfer fluid
IHE	intermediate heat exchanger
LMTD	logarithmic mean temperature difference
MOPSO	multi-objective particle swarm optimizer
ORC	organic Rankine cycle
**Subscripts**	
0	reference state
1,2,…,i	system state points
*con*	condenser
*D*	destruction
*dusp*	desuperheater
*eva*	evaporator
*exh*	exhaust gas
*F*	fuel
*in*	inlet
*k*	*k*th component
*out*	outlet
*P*	product
*p*	pump
*pre*	preheater
*sys*	system
*t*	turbine
*w*	water
*wf*	working fluid
**Greek letters**	
ε	exergy efficiency (%)
ƞ	isentropic efficiency(%)
ρ	density (kg/m^3^)
ω	life cycle inventory associated with the production of 1 kg of material (mpts/kg)
δ	thickness (m)
n	lifetime of the system (year)
τ	annual plant operation with full capacity (h)
